# Comparison between the new Xpert *Mycobacterium tuberculosis/*Rifampicin (MTB/RIF) Ultra assay and Xpert MTB/RIF for diagnosis of extra-pulmonary tuberculosis in a tertiary care center

**DOI:** 10.1128/spectrum.01647-25

**Published:** 2025-11-10

**Authors:** Aroop Mohanty, Atul R. Rukadikar, Vivek Hada, Parul Singh, Subodh Kumar Pandey, Mahima Mittal, Gaurav Gupta, Kanishka Kumar, Rama Shankar Rath, U. Venketesh, Amresh Kumar Singh, Kumari Neha Singh, Rachana Mehta, Sanjit Sah, Shriyansh Srivastava, Vasso Apostolopoulos

**Affiliations:** 1 Department of Microbiology, AIl India Institute of Medical Sciences 560851, Gorakhpur, Uttar Pradesh, India; 2 Department of Pulmonary Medicine, AIl India Imnstitute of Medical Sciences 560851, Gorakhpur, Uttar Pradesh, India; 3 Department of Paediatrics, AIl India Institue of Medical Sciences 560851, Gorakhpur, Uttar Pradesh, India; 4 Department of General Surgery, AIl India Institute of Medical Sciences 560851, Gorakhpur, Uttar Pradesh, India; 5 Department of General Medicine, AIl India Institute of Medical Sciences 560851, Gorakhpur, Uttar Pradesh, India; 6 Department of Community and Family Medicine, AIl India Institute of Medical Sciences 560851, Gorakhpur, Uttar Pradesh, India; 7 Associate Professor and Nodal/In-charge IRL, Department of Microbiology, BRD Medical College 30063 , Gorakhpur, Uttar Pradesh, India; 8 Dr. Lal Path Labs, Nepal - Chandol-4, Kathmandu, Nepal; 9 Clinical Microbiology, RDC, Manav Rachna International Institute of Research and Studies, Faridabad, Haryana, India; 10 Department of Pediatrics, Dr. D.Y. Patil Medical College, Hospital and Research Centre. Dr. D.Y. Patil Vidyapeeth (Deemed-to-be-University), Pune, Maharashtra, India; 11 Department of Public Health Dentistry, Dr. D.Y. Patil Dental College and Hospital, Dr. D.Y. Patil Vidyapeeth, Pune, Maharashtra, India; 12 Department of Medicine, Korea University, Seoul, South Korea; 13 Department of Pharmacy, School of Medical and Allied Sciences, Galgotias University 357911, Greater Noida, Uttar Pradesh, India; 14 School of Health and Biomedical Sciences, RMIT University 5376, Melbourne, Victoria, Australia; Institut National de Santé Publique du Québec, Sainte-Anne-de-Bellevue, Quebec, Canada

**Keywords:** extra-pulmonary tuberculosis, EPTB, India, *Mycobacterium tuberculosis*, MTB, tuberculosis, rifampicin, Xpert MTB/RIF, Xpert MTB/RIF Ultra, Ziehl-Neelsen stain

## Abstract

Tuberculosis remains a significant global health challenge, with the re-emergence of *Mycobacterium tuberculosis* (MTB) posing serious concerns, especially in its extra-pulmonary forms. Rapid and accurate diagnosis is crucial for effective management. This study evaluates the performance of the Xpert MTB/RIF Ultra assay compared with the Xpert MTB/RIF and traditional microscopy (Ziehl-Neelsen stain) in diagnosing extra-pulmonary tuberculosis (EPTB) in a tertiary care center in northern India. A total of 100 samples collected from September 2024 to February 2025 underwent testing using these methods. Our findings indicate that the Xpert MTB/RIF Ultra assay demonstrated superior sensitivity and reliability compared with the Xpert MTB/RIF, whereas microscopy was the least effective. This study is the first of its kind in India, highlighting the enhanced diagnostic capabilities of the Xpert MTB/RIF Ultra assay for EPTB.

**IMPORTANCE:**

This is the first study to come out of India where the newly launched Xpert Ultra/RIF test has been used to detect Paucibacillary EPTB cases in comparison to the old Xpert MTB/RIF test.

## INTRODUCTION

Tuberculosis (TB) remains a global threat despite the advent of new diagnostics and therapeutics. TB has returned to being the world’s leading cause of death from a single infectious disease after the deadly COVID-19 pandemic since 2020, marked by ongoing outbreaks. Over 10 million people die from TB annually, and the numbers have been rising since 2021. According to the World Health Organization (WHO)^1^ Global tuberculosis report 2024, 10.8 million people developed TB in the year 2023 (1), most being from South-East Asia (45%) and Africa (24%). Of the total number of cases, 87% belong to the top 30 high TB burden countries, with India (26%) among the five countries contributing to 56% of the total. With this high burden of TB in the country, the mainstay of diagnostics used to detect the tubercle bacilli is molecular-based assays.

Tuberculosis outside the lung tissue is called extrapulmonary tuberculosis (EPTB), and this accounts for 15% of all reported cases (2, 3). Most of the EPTB samples are paucibacillary in nature with low bacterial load, making their diagnosis difficult using conventional methods. In the majority of the cases, treatment for TB is initiated based on clinical judgment, exposure history, clinical symptoms, and radiological investigations. This diagnostic gap was somewhat filled following the introduction of the Xpert MTB/RIF (Cepheid, Sunnyvale, CA, USA) assay in December 2010 by the WHO. Xpert MTB/RIF is the first rapid 2 h point-of-care molecular test for *Mycobacterium tuberculosis* (MTB) and resistance to the antibiotic rifampicin (RIF), which was later introduced into the National Tuberculosis Elimination Program (NTEP) in India (4). However, despite its excellent performance in smear-positive sputum samples, the Xpert MTB/RIF had limited sensitivity in smear-negative samples and those with a low bacilli load (5). It also gave variable results when tested on different types of extra-pulmonary samples (6).

However, to overcome these limitations, in 2017, the technology was improved with the launch of Xpert MTB/RIF Ultra assay, a more rapid and improved sensitivity test with lower limit of detection (LOD: 15.6 CFU/mL) than Xpert (LOD: 112.6 CFU/mL) owing to the use of multi-copy insertion elements (IS1081 and IS6110) as MTB targets and gives an additional semi-quantitative “trace” result (7, 8). It gives excellent results in smear-negative patients, in cases with low bacterial loads, and for patients with human immunodeficiency virus–tuberculosis co-infections.

Most of the studies on Xpert MTB/RIF Ultra in India have been conducted in adults with pulmonary TB. To date, the NTEP uses the Xpert MTB/RIF cartridge. We conducted the study to compare the Xpert MTB/RIF Ultra assay with the earlier molecular assays, namely Xpert MTB/RIF, to detect EPTB in various types of samples from a tertiary care teaching hospital in Northern India.

## MATERIALS AND METHODS

### Study design and setting

This is a secondary data analysis of the available laboratory records of patients, conducted between September 2024 and February 2025 in a 750-bed tertiary care center located in the state of Uttar Pradesh, India. The records of the patients were extracted in the specified time period from the various data sources. Samples were collected from clinically suspected patients of EPTB and were subjected to testing first by conventional microscopy and then by Xpert MTB/RIF and Xpert MTB/RIF Ultra assays.

### Study population and data source

The study population comprised all individuals with clinically suspected EPTB based on the clinical symptoms who visited the All-India Institute of Medical Sciences (AIIMS), Gorakhpur, during the study period. The samples were collected from various sites and sent to the microbiology laboratory of AIIMS Gorakhpur for evaluation.

### Study tools

The study utilized multiple diagnostic tests, including Microscopy (Ziehl-Neelsen stain), Xpert MTB/RIF, and Xpert MTB/RIF Ultra. Additionally, a structured patient register form was employed to systematically record relevant data.

### Diagnostic methods

#### Microscopy

All the samples were first examined grossly for quality of the specimen and then stained using the Ziehl-Nelsen staining procedure, and the results were reported as per the NTEP guidelines.

#### Xpert MTB/RIF and Xpert MTB/RIF Ultra

The sample reagent buffer, containing NaOH and isopropanol in a 2:1 ratio, was added, mixed, and then kept for incubation at room temperature for 15 min. The clinical sample (2 mL) was then transferred to either the Xpert MTB/RIF or Xpert MTB/RIF Ultra cartridge and loaded onto the respective instrument. The instrument performs all steps automatically. It is based upon the hemi-nested real-time PCR amplification principle and detects the target region in approximately 2 h. Both the diagnostic modalities detect MTB as well as Rifampicin resistance if present in the pathogen.

### Statistical analysis

The data collected from the records were entered in Microsoft Excel and cleaned before analysis. Analysis was performed using STATA-17 (College Station, Texas, USA). Descriptive, agreement, and diagnostic performance analyses were conducted. Positive TB case detection rates were calculated with 95% confidence intervals (CIs). Agreement analysis employed Kappa statistics to assess concordance between diagnostic tests. Diagnostic performance measures, including sensitivity, specificity, positive predictive value (PPV), negative predictive value (NPV), and overall accuracy, were computed using the Composite Reference Standard (CRS) as the gold standard. The receiver operating characteristic (ROC) curve analysis was performed to compare the diagnostic accuracy. The CRS is constructed based on a combination of results from Microscopy (Ziehl-Neelsen stain), Xpert MTB/RIF, and Xpert MTB/RIF Ultra. A participant is classified as EPTB-positive under CRS if at least two of the tests yield positive results (9). This approach enhances diagnostic reliability by integrating multiple testing modalities.

## RESULTS

Over a time period of 6 months between September 2024 and February 2025, a total of 100 EPTB samples were tested using first the conventional Ziehl-Neelsen microscopy and then subsequently by both Xpert MTB/RIF and Xpert MTB/RIF Ultra for MTB. Among the samples, 37% belonged to the 1–25 age group, followed by 33% in the 26–49 age group and 30% in the 50+ age group (Table 1). The majority of the sampled population was males (62%), followed by females (38%). Of the total samples, pleural fluid (25/100) and pus (25/100) contributed to 50%, whereas bronchoalveolar lavage (20/100) and gastric aspirates (15/100), along with others, made up the remaining (Table 1).

**TABLE 1:** Sample demographic characteristics

	Number of samples	Percent
Age		
1–25	37	37
26–49	33	33
50+	30	30
Sex		
Female	38	38
Male	62	62
Sample type		
Pleural fluid	25	25
Bronchoalveolar lavage fluid	20	20
Gastric aspirate	15	15
Pus	25	25
Ascites fluid	8	8
Other	7	7
Total number of samples	100	

Among the 100 samples, conventional microscopy (Ziehl-Nelsen Stain) detected one as positive for the presence of acid-fast bacilli (1%; 95% CI: 0.1, 7.0). However, the Xpert MTB/RIF detected MTB in 16/100 samples (16%; 95% CI: 10.0, 24.7) (Table 2). Among these 16 positive samples, seven were classified as medium, seven as low, and two as very low. The RIF resistance was detected in 1/16 positive samples that had detected MTB load as the medium. In comparison, the Xpert MTB/RIF Ultra detected MTB in 31/100 samples (95% CI: 22.6, 40.9) (Table 2). Of the 31 samples, 3 were classified as high, 3 as medium, 10 as low, 4 as very low, and 11 in the trace category. All samples detected positive for MTB under the Trace category showed RIF as indeterminate. Only 2/31 positive samples on Xpert MTB/RIF Ultra were positive for RIF resistance.

**TABLE 2:** Patterns of *M. tuberculosis* detection across three diagnostic tests

	Number of tests	Percent	95% CI
Microscopy Ziehl-Neelsen Stain			
Negative	99	99	93, 99.9
Positive	1	1	0.1, 7.0
Xpert MTB/RIF			
Not detected	84	84	75.3, 90
Detected	16	16	10, 24.7
Xpert MTB/RIF Ultra			
Not detected	69	69	59.1, 77.4
Detected	31	31	22.6, 40.9

As far as the agreement was concerned between the tests, conventional microscopy (Ziehl-Neelsen Stain) showed poor agreement with Xpert MTB/RIF (kappa = 0.1007, 85% agreement, *P* = 0.011) and Xpert MTB/RIF Ultra (kappa = 0.044, 70% agreement, *P* = 0.067). The pattern was almost similar between Xpert MTB/RIF and Xpert MTB/RIF Ultra (kappa = 0.5955, 85% agreement, *P* ≤ 0.001). Table 3 shows a substantial variation in concordance levels among MTB diagnostic tests.

**TABLE 3:** Kappa agreement analysis between tuberculosis diagnostic tests

	Agreement	Expected agreement	Kappa	*P*-value
Microscopy Ziehl-Neelsen Stain vs. Xpert MTB/RIF	85.00%	83.32%	0.1007	0.011
Microscopy Ziehl-Neelsen Stain vs. Xpert MTB/RIF Ultra	70.00%	68.62%	0.044	0.067
Xpert MTB/RIF MTB vs. Xpert MTB/RIF Ultra	85.00%	62.92%	0.5955	≤ 0.001

The diagnostic performance of the three MTB detection methods, microscopy (Ziehl-Neelsen Stain), Xpert MTB/RIF, and Xpert MTB/RIF Ultra, was assessed by considering the CRS as a gold standard (Table 4). Microscopy demonstrated poor sensitivity (3.0%, 95% CI: 0.2–30.2) but perfect specificity (100%, 95% CI: 95.7–100), leading to an accuracy of 68% (95% CI: 58.9–77.1). In contrast, Xpert MTB/RIF Ultra exhibited the best diagnostic efficacy, with a high sensitivity of 93.9% (95% CI: 79.8–99.3) and perfect specificity (100%, 95% CI: 94.6–100), achieving an accuracy of 98% (95% CI: 93.0–99.8). Notably, all tests with perfect specificity had a positive predictive value of 100%, ensuring no false-positive diagnoses, whereas Xpert MTB/RIF Ultra had the highest negative predictive value (97.1%, 95% CI: 89.9–99.6), showing it to be the most reliable test for detecting MTB cases.

**TABLE 4:** Sensitivity and specificity analyses considering CRS as the reference category

Measures	Percent	95% CI
Microscopy Ziehl-Neelsen Stain		
Sensitivity	3.0	0.2, 30.2
Specificity	100	95.7, 100
Positive predictive value	100	2.5, 100
Negative predictive value	67.7	57.5-76.7
Accuracy	68	58.9, 77.1
Observations (*n*, number)	(*n* = 100)	
Xpert MTB/RIF		
Sensitivity	48.5	30.8, 66.5
Specificity	100	94.6, 100
Positive predictive value	100	79.4, 100
Negative predictive value	79.8	69.6, 87.7
Accuracy	83	75.1, 89.3
Observations (*n*, number)	(*n* = 100)	
Xpert MTB/RIF Ultra		
Sensitivity	93.9	79.8, 99.3
Specificity	100	94.6, 100
Positive predictive value	100	88.8, 100
Negative predictive value	97.1	89.9, 99.6
Accuracy	98	93, 99.8
Observations (*n*, number)	(*n* = 100)	

**Fig 1:**
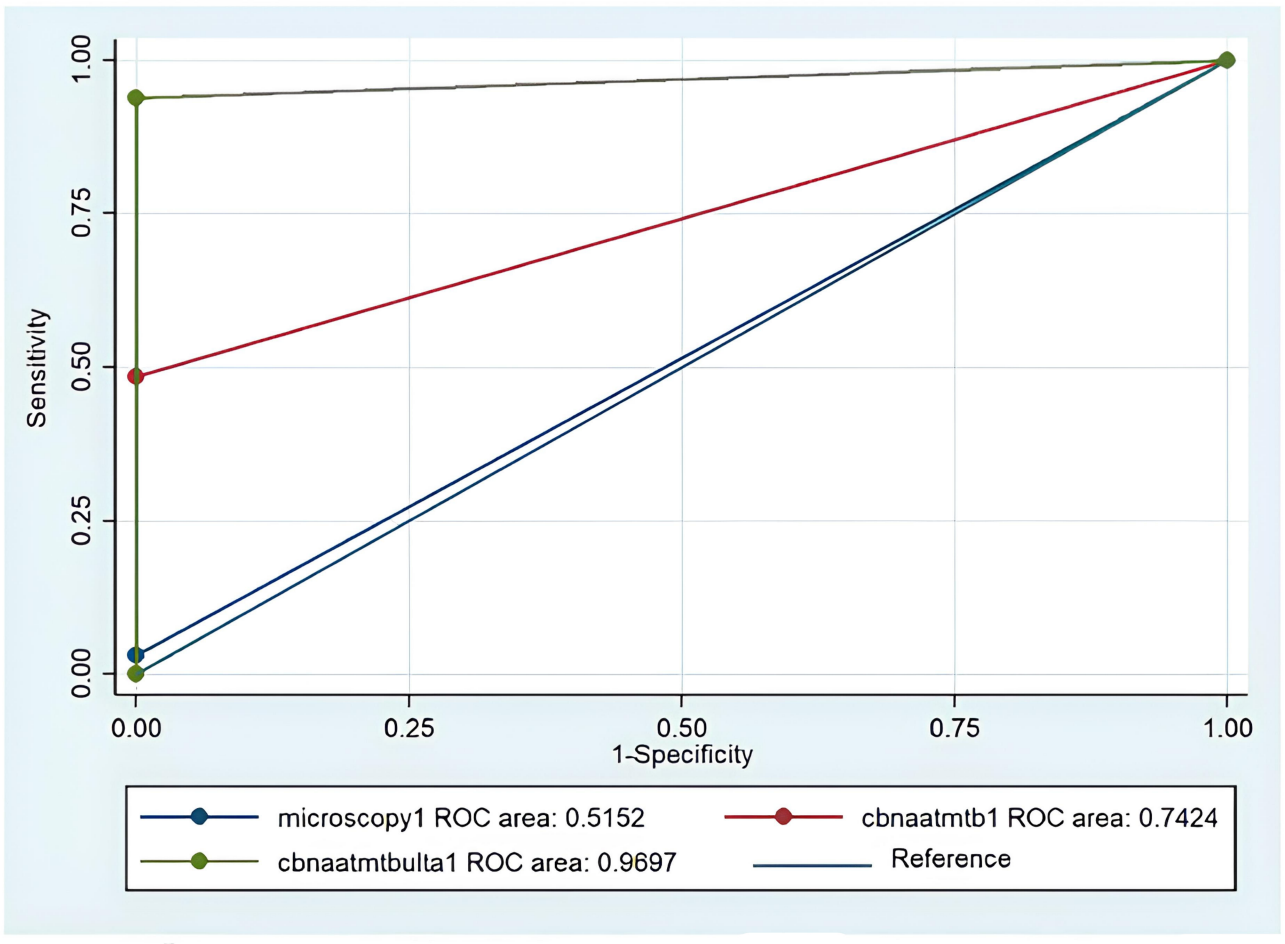
ROC curve of different *M. tuberculosis* (MTB) diagnostic tests considering the CRS as reference.

Figure 1 displays the ROC curve of different MTB diagnostic tests considering the CRS as the gold standard. The highest diagnostic accuracy observed in cartridge-based nucleic acid amplification test (CBNAAT) of the Xpert MTB/RIF Ultra (AUC = 0.9697). Microscopy (Ziehl-Neelsen Stain) performed poorly (AUC = 0.5152), with very low sensitivity. Overall, CBNAAT using the Xpert MTB/RIF Ultra version of the test is the most effective diagnostic tool, whereas microscopy is the least reliable, performing only slightly better than random chance.

## DISCUSSION

Herein we evaluated the correlation between the improved Xpert MTB/RIF Ultra assay with that of the Xpert MTB/RIF, which is now mostly available in most of the medical colleges and hospitals in India for the detection of EPTB. Both of the above tests work on the basis of amplifying the nucleic acids and therefore improving their detectability and identifying the pathogenic organism (6). The Xpert MTB/RIF Ultra assay to date has been approved by the WHO for the diagnosis of both pulmonary as well as EPTB; however, in India, to date, it has been recommended to only be used for unprocessed sputum samples or concentrated sediments prepared from induced or expectorated sputum. Although Xpert MTB/RIF performs well in most cases of TB, its performance is not as good in paucibacillary conditions of EPTB (10).

Our study is unique in nature, as it is the first study from India to evaluate both Xpert MTB/RIF and Xpert MTB/RIF Ultra and compare them for the detection of EPTB. Here, we included 100 extra-pulmonary samples with interpretable results in all 100 of them. Despite the limited sample size of the study, we have identified significant findings. As per the Global Tuberculosis Report 2024, the disease affects men more than women, and a similar finding was also observed in our study with 62% of the total being males.

Of the total 100 extra-pulmonary samples included and tested by conventional microscopy and both by molecular assays, the Xpert MTB/RIF Ultra detected *M. tuberculosis* complex in 16 additional cases compared with the Xpert MTB/RIF. Conventional Ziehl-Neelsen microscopy played a limited role, detecting acid-fast bacilli in 1/100 samples, which was graded as Scanty. The improved sensitivity and specificity of Xpert MTB/RIF Ultra versus Xpert MTB/RIF (93.4% vs 48.5%) led to a more significant proportion of microbiologically confirmed EPTB. These findings are similar to those of Wu et al. (11), who also found improved sensitivity for Xpert MTB/RIF Ultra (83.7%) compared with Xpert MTB/RIF (67.4%). Our study illustrates a highly significant difference in sensitivity of more than 45% between Xpert MTB/RIF and Xpert MTB/RIF Ultra, which can be very well explained by the greater ability in view of the lower detection threshold of Xpert MTB/RIF Ultra. The improved sensitivity of Xpert MTB/RIF Ultra is due to the use of multi-copy *IS1081* and *IS6110* insertion elements specific to MTB as target sequences, allowing the detection of samples with lowest bacilli load (8, 12). Consistently, 15 extrapulmonary specimens with negative Xpert MTB/RIF results showed positive results in the Xpert MTB/RIF Ultra assay, demonstrating an improved EPTB detection. This is of utmost importance in extra-pulmonary samples, which are paucibacillary in nature and are very difficult to diagnose.

Second, out of the 31 samples that were detected positive for MTB-complex on Xpert MTB/RIF Ultra, 11 were classified as trace. These included a substantial amount of pleural fluid (45%) along with bronchoalveolar lavage and pus samples, illustrating that clinicians need to be careful when dealing with such patients. This is an additional semi-quantitative category that was not present in the Xpert MTB/RIF. All these 11 samples when tested for RIF resistance gave indeterminate results, emphasizing the further need for drug susceptibility testing. Furthermore, of the 31 positive samples of Xpert MTB/RIF Ultra, only two samples demonstrated rifampicin resistance—one each with a low and high level of MTB complex. The use of Xpert MTB/RIF Ultra could reduce the dependency on multiple diagnostic modalities (e.g., microscopy and culture), thereby shortening the diagnostic timeline and reducing costs related to prolonged illness and empirical treatments. However, programmatic integration would require training, awareness, and logistical upgrades in peripheral settings.

### Limitations

Since the data were obtained from a single hospital with a limited number of samples, generalizing the findings may be challenging. In addition, the various types of EPTB samples were very few in number, especially those of peritoneal fluid, urine, and pericardial fluid. Larger multi-center studies, with larger sample sizes, will be required to further evaluate the performance of Xpert MTB/RIF Ultra on such samples. Additionally, variations in test conditions and operator expertise could influence results, potentially affecting diagnostic accuracy. In terms of rifampicin resistance determination, all samples with trace levels of MTB-complex on Xpert MTB/RIF Ultra need to be sent for further testing, as they will give indeterminate results.

## Data Availability

All data are available within the article. Remaining data can be obtained from the corresponding author on reasonable request.

## References

[1] Global tuberculosis report 2024. 2024. https://www.who.int/teams/global-programme-on-tuberculosis-and-lung-health/tb-reports/global-tuberculosis-report-2024.

[2] Koenig SP, Furin J. 2016. Update in tuberculosis/pulmonary infections 2015. Am J Respir Crit Care Med 194:142–146. doi:10.1164/rccm.201601-0129UP 27420359 PMC5003219

[3] Ketata W, Rekik WK, Ayadi H, Kammoun S. 2015. Extrapulmonary tuberculosis. Rev Pneumol Clin 71:83–92. doi:10.1016/j.pneumo.2014.04.001 25131362

[4] WHO Guidelines Approved by the Guidelines Review Committee. 2014. Xpert MTB/RIF implementation manual: technical and operational “How-To”; practical considerations. Geneva World Health Organization 25473699

[5] Steingart KR, Schiller I, Horne DJ, Pai M, Boehme CC, Dendukuri N. 2014. Xpert MTB/RIF assay for pulmonary tuberculosis and rifampicin resistance in adults. Cochrane Database Syst Rev 2014:CD009593. doi:10.1002/14651858.CD009593.pub3 24448973 PMC4470349

[6] Huo ZY, Peng L. 2018. Is Xpert MTB/RIF appropriate for diagnosing tuberculous pleurisy with pleural fluid samples? A systematic review. BMC Infect Dis 18:284. doi:10.1186/s12879-018-3196-4 29940951 PMC6019837

[7] Opota O, Mazza-Stalder J, Greub G, Jaton K. 2019. The rapid molecular test Xpert MTB/RIF ultra: towards improved tuberculosis diagnosis and rifampicin resistance detection. Clin Microbiol Infect 25:1370–1376. doi:10.1016/j.cmi.2019.03.021 30928564

[8] Chakravorty S, Simmons AM, Rowneki M, Parmar H, Cao Y, Ryan J, Banada PP, Deshpande S, Shenai S, Gall A, Glass J, Krieswirth B, Schumacher SG, Nabeta P, Tukvadze N, Rodrigues C, Skrahina A, Tagliani E, Cirillo DM, Davidow A, Denkinger CM, Persing D, Kwiatkowski R, Jones M, Alland D. 2017. The new Xpert MTB/RIF Ultra: improving detection of Mycobacterium tuberculosis and resistance to rifampin in an assay suitable for point-of-care testing. mBio 8:e00812-17. doi:10.1128/mBio.00812-17 28851844 PMC5574709

[9] Kaur H, Guglani V, Singhal L, Randev S, Kumar P, Gupta V. 2023. The new Xpert Mycobacterium tuberculosis/rifampicin (MTB/Rif) Ultra assay in comparison to Xpert MTB/Rif assay for diagnosis of tuberculosis in children and adolescents. J Trop Pediatr 70:fmad046. doi:10.1093/tropej/fmad046 38116810

[10] Chitnis AS, Davis JL, Schecter GF, Barry PM, Flood JM. 2015. Review of nucleic acid amplification tests and clinical prediction rules for diagnosis of tuberculosis in acute care facilities. Infect Control Hosp Epidemiol 36:1215–1225. doi:10.1017/ice.2015.145 26166303

[11] Wu X, Tan G, Gao R, Yao L, Bi D, Guo Y, Yu F, Fan L. 2019. Assessment of the Xpert MTB/RIF Ultra assay on rapid diagnosis of extrapulmonary tuberculosis. Int J Infect Dis 81:91–96. doi:10.1016/j.ijid.2019.01.050 30738907

[12] Sankar S, Kuppanan S, Balakrishnan B, Nandagopal B. 2011. Analysis of sequence diversity among IS6110 sequence of Mycobacterium tuberculosis: possible implications for PCR based detection. Bioinformation 6:283–285. doi:10.6026/97320630006283 21738331 PMC3124695

